# Supplementary material for: Genetic Polymorphisms of IGF1 and IGF1R Genes and Their Effects on Growth Traits in Hulun Buir Sheep
Source: Genes (Basel). 2022 Apr 9;13(4):666. doi: 10.3390/genes13040666 (PMC9031115; doi:10.3390/genes13040666)
Supplement: Supplementary file 1 [file genes-13-00666-s001.zip › Table S2.pdf]

Table S2. Associations for the SNPs of *IGF1* gene with body weight traits and ADG traits in Hulun Buir sheep (mean ± SE, n = 229)

SNPs	Genotypes	Body weight (kg)			Average daily gain (ADG) (g)		
		BW	WW	NBW	0-4 ADG	4-9 ADG	0-9 ADG
SNP1	GG (225)	4.21 ± 0.04	23.32 ± 0.47	32.36 ± 0.50	153.35 ± 3.47	62.19 ± 1.27	104.47 ± 1.76
	GA (4)	4.70 ± 0.16	30.48 ± 1.82	39.60 ± 0.83	200.75 ± 12.96	63.66 ± 9.52	128.41 ± 2.27
SNP2	TT (113)	4.21 ± 0.06	22.80 ± 0.67	31.96 ± 0.73	150.49 ± 5.08	63.21 ± 1.56	103.48 ± 2.61
	TC (102)	4.23 ± 0.07	24.33 ± 0.70	33.35 ± 0.72	160.02 ± 5.00	61.74 ± 2.20	107.57 ± 2.48
	CC (14)	4.24 ± 0.14	22.33 ± 1.72	30.79 ± 1.85	143.53 ± 12.40	58.39 ± 3.91	98.07 ± 6.41
SNP3	GG (147)	4.22 ± 0.06	23.02 ± 0.59	32.09 ± 0.62	151.10 ± 4.34	**62.22 ± 1.46**^{a}	103.43 ± 2.16
	GA (76)	4.26 ± 0.07	24.17 ± 0.79	33.38 ± 0.87	159.16 ± 5.92	**63.70 ± 2.45**^{a}	108.05 ± 3.10
	AA (6)	3.78 ± 0.23	24.45 ± 2.26	30.52 ± 2.56	166.58 ± 15.09	**42.17 ± 4.83**^{b}	99.88 ± 8.60

BW = birth weight; WW = Weaning weight; NBW = weight at 9-mongth of age; 0-4 ADG, 4-9 ADG and 0-9 ADG represent the average daily weight gain before weaning, after weaning and from birth to 9-month of age, respectively. Different letter (small letters: $p < 0.05$; capital letters: $p < 0.01$) superscripts with boldface font in a column indicate significant differences among the different genotypes.
