# Supplementary material for: Genetic Polymorphisms of IGF1 and IGF1R Genes and Their Effects on Growth Traits in Hulun Buir Sheep
Source: Genes (Basel). 2022 Apr 9;13(4):666. doi: 10.3390/genes13040666 (PMC9031115; doi:10.3390/genes13040666)
Supplement: Supplementary file 1 [file genes-13-00666-s001.zip › Table S3.pdf]

Table S3. Associations for the SNPs of *IGF1* gene with body size traits in Hulun Buir sheep (mean ± SE, n = 229)

SNPs	Genotypes	Weaned body size (cm)			Body size of 9-month (cm)		
		WBH	WBL	WCG	NBH	NBL	NCG
SNP1	GG (225)	56.04 ± 0.36	57.20 ± 0.46	**68.30 ± 0.49**^{b}	63.82 ± 0.29	**66.71 ± 0.34**^{b}	83.27 ± 0.53
	GA (4)	59.15 ± 1.09	61.25 ± 1.71	**76.13 ± 1.98**^{a}	63.38 ± 2.76	**72.38 ± 1.97**^{a}	88.38 ± 1.14
SNP2	TT (113)	55.62 ± 0.50	56.69 ± 0.69	67.71 ± 0.70	63.72 ± 0.42	66.56 ± 0.50	**82.75 ± 0.80**^{ab}
	TC (102)	56.88 ± 0.52	58.14 ± 0.63	69.32 ± 0.70	63.82 ± 0.43	67.14 ± 0.51	**84.39 ± 0.73**^{a}
	CC (14)	54.50 ± 1.37	55.89 ± 1.43	67.97 ± 1.96	63.78 ± 1.18	66.25 ± 1.35	**80.69 ± 1.95**^{b}
SNP3	GG (147)	55.94 ± 0.46	56.93 ± 0.60	67.98 ± 0.61	63.68 ± 0.38	66.41 ± 0.43	82.86 ± 0.66
	GA (76)	56.31 ± 0.58	57.80 ± 0.69	69.19 ± 0.84	64.01 ± 0.47	67.56 ± 0.61	84.13 ± 0.92
	AA (6)	57.08 ± 0.61	58.83 ± 1.45	70.08 ± 3.05	63.00 ± 1.03	66.17 ± 1.38	83.83 ± 3.60

WBH, WBL and WCG represent the body height, body length and chest girth measured at weaning respectively; NBH, NBL and NCG represent the body height, body length and chest girth measured at 9-mongth of age respectively. Different letter (small letters: $p < 0.05$; capital letters: $p < 0.01$) superscripts with boldface font in a column indicate significant differences among the different genotypes.
