# Supplementary material for: Genetic Polymorphisms of IGF1 and IGF1R Genes and Their Effects on Growth Traits in Hulun Buir Sheep
Source: Genes (Basel). 2022 Apr 9;13(4):666. doi: 10.3390/genes13040666 (PMC9031115; doi:10.3390/genes13040666)
Supplement: Supplementary file 1 [file genes-13-00666-s001.zip › Table S4.pdf]

Table S4. Associations for the SNPs of *IGF1R* gene with body weight traits and ADG traits in Hulun Buir sheep (mean ± SE, n = 229)

SNPs	Genotypes	Body weight (kg)			Average daily gain (ADG) (g)		
		BW	WW	NBW	0-4 ADG	4-9 ADG	0-9 ADG
SNP4	CC (227)	4.20 ± 0.04	23.31 ± 0.47	32.31 ± 0.50	153.12 ± 3.45	62.10 ± 1.24	104.33 ± 1.76
	CT (2)	4.04 ±31.00	27.10 ± 5.70	33.80 ± 5.10	190.28 ± 39.21	46.33 ± 3.67	112.19 ± 17.23
SNP5	GG (187)	4.18 ± 0.05	23.40 ± 0.53	32.35 ± 0.55	153.42 ± 3.90	61.72 ± 1.28	104.35 ± 1.93
	GA (42)	4.28 ± 0.10	23.10 ± 0.94	32.21 ± 1.13	153.58 ± 7.24	63.04 ± 3.53	104.62 ± 4.12
SNP6	TT (71)	4.15 ± 0.08	23.43 ± 0.85	32.32 ± 0.90	154.48 ± 6.43	61.37 ± 2.03	104.48 ± 3.20
	TC (104)	4.18 ± 0.07	22.60 ± 0.66	31.59 ± 0.73	147.75 ± 4.76	61.90 ± 1.90	101.70 ± 2.52
	CC (54)	4.28 ± 0.09	24.68 ± 1.00	33.75 ± 1.03	163.06 ± 7.50	62.86 ± 2.62	109.48 ± 3.65
SNP7	CC (83)	4.16 ± 0.08	4.16 ± 0.08	31.97 ± 0.82	152.55 ± 5.70	61.21 ± 2.04	103.38 ± 2.92
	CT (114)	4.21 ± 0.06	4.21 ± 0.06	32.09 ± 0.71	151.35 ± 4.79	62.00 ± 1.74	103.48 ± 2.47
	TT (32)	4.22 ± 0.12	4.22 ± 0.12	34.10 ± 1.32	163.23 ± 9.95	63.74 ± 3.41	110.30 ± 4.55
SNP8	TT (74)	**4.24 ± 0.08^{a}**	**25.14 ± 0.81^{A}**	**33.92 ± 0.84^{A}**	**165.56 ± 5.81^{A}**	60.72 ± 2.00	**109.61 ± 2.86^{A}**
	TC (118)	**4.25 ± 0.06^{a}**	**23.14 ± 0.64^{A}**	**32.41 ± 0.70^{A}**	**152.11 ± 4.85^{A}**	63.94 ± 1.86	**104.74 ± 2.50^{A}**
	CC (37)	**3.95 ± 0.12^{b}**	**20.44 ± 1.08^{B}**	**28.87 ± 1.13^{B}**	**133.49 ± 7.99^{B}**	58.12 ± 2.54	**92.88 ± 4.00^{B}**
SNP9	GG (184)	4.18 ± 0.05	23.35 ± 0.54	32.31 ± 0.56	152.96 ± 3.94	61.83 ± 1.29	55.85 ± 0.41
	GA (45)	4.26 ± 0.10	23.35 ± 0.90	32.38 ± 1.09	155.43 ± 6.89	62.48 ± 3.39	56.83 ± 0.69
SNP10	AA (158)	4.28 ± 0.05	24.15 ± 0.54	33.00 ± 0.59	158.89 ± 4.00	61.11 ± 1.50	106.49 ± 2.07
	AG (65)	4.02 ± 0.08	21.29 ± 0.89	30.60 ± 0.93	139.19 ± 6.71	64.15 ± 2.31	98.89 ± 3.32
	GG (6)	3.95 ± 0.24	24.45 ± 2.99	33.23 ± 3.31	164.55 ± 22.22	60.63 ± 3.89	108.89 ± 11.99
SNP11	TT (167)	4.15 ± 0.05	23.11 ± 0.56	32.04 ± 0.59	151.13 ± 4.12	61.61 ± 1.39	103.25 ± 2.05
	TC (57)	4.33 ± 0.09	24.09 ± 0.86	33.14 ± 0.97	160.45 ± 6.58	62.54 ± 2.73	107.63 ± 3.52
	CC (5)	4.09 ± 0.36	22.82 ± 1.96	32.50 ± 2.92	151.01 ± 16.03	66.95 ± 7.43	105.73 ± 10.92
SNP12	CC (213)	4.19 ± 0.05	23.37 ± 0.48	32.36 ± 0.52	153.54 ± 3.56	62.10 ± 1.30	104.54 ± 1.81
	CT (16)	4.26 ± 0.21	23.06 ± 1.84	31.80 ± 1.86	152.21 ± 13.89	60.13 ± 3.33	102.56 ± 6.68
SNP13	CC (113)	4.27 ± 0.06	24.02 ± 0.67	**33.23 ± 0.72^{a}**	158.82 ± 5.01	63.55 ± 1.89	**107.62 ± 2.57^{a}**
	CG (95)	4.13 ± 0.07	23.00 ± 0.71	**31.87 ± 0.73^{ab}**	150.58 ± 5.15	61.33 ± 1.81	**102.84 ± 2.53^{ab}**
	GG (21)	4.04 ± 0.16	21.31 ± 1.59	**29.48 ± 1.71^{b}**	137.44 ± 11.44	56.27 ± 2.95	**94.11 ± 5.87^{b}**

BW = birth weight; WW = Weaning weight; NBW = weight at 9-mongth of age; 0-4 ADG, 4-9 ADG and 0-9 ADG represent the average daily weight gain before weaning, after weaning and from birth to 9-month of age, respectively. Different letter (small letters: $p < 0.05$; capital letters: $p < 0.01$) superscripts with boldface font in a column indicate significant differences among the different genotypes.
