# Supplementary material for: Genetic Polymorphisms of IGF1 and IGF1R Genes and Their Effects on Growth Traits in Hulun Buir Sheep
Source: Genes (Basel). 2022 Apr 9;13(4):666. doi: 10.3390/genes13040666 (PMC9031115; doi:10.3390/genes13040666)
Supplement: Supplementary file 1 [file genes-13-00666-s001.zip › Table S5.pdf]

Table S5. Associations for the SNPs of *IGF1R* gene with body size traits in Hulun Buir sheep (mean ± SE, n = 229)

SNPs	Genotypes	Weaned body size (cm)			Body size of 9-month (cm)		
		WBH	WBL	WCG	NBH	NBL	NCG
SNP4	CC (227)	56.02 ± 0.36	57.22 ± 0.46	68.39 ± 0.50	63.71 ± 0.29	66.80 ± 0.34	83.35 ± 0.52
	CT (2)	58.50 ± 1.50	60.25 ± 1.75	73.00 ± 4.00	64.00 ± 0.01	69.00 ± 2.00	81.50 ± 2.50
SNP5	GG (187)	55.92 ± 0.40	57.17 ± 0.53	68.50 ± 0.56	63.80 ± 0.32	66.62 ± 0.37	83.35 ± 0.58
	GA (42)	56.61 ± 0.72	57.58 ± 0.84	68.11 ± 0.97	63.32 ± 0.61	67.70 ± 0.87	83.25 ± 1.13
SNP6	TT (71)	55.82 ± 0.61	57.27 ± 0.75	68.80 ± 0.91	64.13 ± 0.54	**67.22 ± 0.57[ab]**	83.15 ± 0.92
	TC (104)	55.61 ± 0.53	56.52 ± 0.72	67.74 ± 0.72	63.59 ± 0.41	**65.89 ± 0.47[b]**	82.54 ± 0.75
	CC (54)	57.19 ± 0.75	58.60 ± 0.92	69.27 ± 1.01	63.40 ± 0.58	**68.07 ± 0.79[a]**	85.07 ± 1.09
SNP7	CC (83)	55.75 ± 0.56	57.11 ± 0.67	68.49 ± 0.81	64.00 ± 0.50	66.90 ± 0.53	82.89 ± 0.86
	CT (114)	56.06 ± 0.50	57.04 ± 0.69	68.06 ± 0.70	63.52 ± 0.37	66.56 ± 0.49	82.95 ± 0.70
	TT (32)	56.77 ± 1.07	58.36 ± 1.30	69.61 ± 1.36	63.63 ± 0.85	67.50 ± 0.99	85.83 ± 1.53
SNP8	TT (74)	**57.24 ± 0.56[A]**	**58.82 ± 0.71[A]**	**69.93 ± 0.84[A]**	**64.49 ± 0.51[A]**	**67.96 ± 0.56[A]**	**85.86 ± 0.9[A]**
	TC (118)	**55.91 ± 0.51[AB]**	**57.33 ± 0.56[A]**	**68.33 ± 0.7[AB]**	**63.89 ± 0.40[A]**	**66.83 ± 0.49[A]**	**82.97 ± 0.69[B]**
	CC (37)	**54.09 ± 0.88[B]**	**53.84 ± 1.55[B]**	**65.76 ± 1.14[B]**	**61.59 ± 0.59[B]**	**64.47 ± 0.70[B]**	**79.39 ± 1.17[C]**
SNP9	GG (184)	55.85 ± 0.41	57.15 ± 0.54	68.44 ± 0.57	63.79 ± 0.33	66.60 ± 0.37	83.28 ± 0.58
	GA (45)	56.83 ± 0.69	57.63 ± 0.80	68.39 ± 0.95	63.39 ± 0.58	67.70 ± 0.81	83.53 ± 1.09
SNP10	AA (158)	56.59 ± 0.42	57.99 ± 0.47	69.09 ± 0.57	64.20 ± 0.35	67.55 ± 0.41	84.32 ± 0.62
	AG (65)	54.68 ± 0.67	55.39 ± 1.08	66.72 ± 0.98	62.63 ± 0.49	64.99 ± 0.59	80.96 ± 0.91
	GG (6)	56.58 ± 2.33	57.67 ± 3.38	69.50 ± 2.93	62.50 ± 1.94	67.33 ± 2.01	83.00 ± 3.74
SNP11	TT (167)	55.70 ± 0.42	56.94 ± 0.58	68.26 ± 0.60	63.62 ± 0.34	66.50 ± 0.39	83.11 ± 0.61
	TC (57)	57.04 ± 0.70	57.97 ± 0.72	69.01 ± 0.89	63.87 ± 0.51	67.77 ± 0.70	84.07 ± 1.06
	CC (5)	56.20 ± 2.18	59.20 ± 1.24	67.60 ± 2.29	64.80 ± 2.94	66.40 ± 2.98	82.20 ± 1.62
SNP12	CC (213)	56.12 ± 0.36	57.35 ± 0.48	68.52 ± 0.51	63.72 ± 0.30	66.88 ± 0.35	83.42 ± 0.53
	CT (16)	55.00 ± 1.52	55.84 ± 1.27	67.28 ± 1.68	63.63 ± 1.11	65.97 ± 1.30	82.13 ± 2.08
SNP13	CC (113)	56.41 ± 0.49	57.67 ± 0.71	**69.37 ± 0.69[a]**	**64.36 ± 0.42[a]**	**67.22 ± 0.49[a]**	**84.28 ± 0.75[a]**
	CG (95)	55.89 ± 0.56	57.28 ± 0.62	**67.93 ± 0.75[ab]**	**63.35 ± 0.4[ab]**	**66.82 ± 0.5[a]**	**82.87 ± 0.77[ab]**
	GG (21)	54.79 ± 1.32	54.79 ± 1.58	**65.64 ± 1.76[b]**	**61.83 ± 1.09[b]**	**64.64 ± 1.21[b]**	**80.29 ± 1.67[b]**

WBH, WBL and WCG represent the body height, body length and chest girth measured at weaning respectively; NBH, NBL and NCG represent the body height, body length and chest girth measured at 9-mongth of age respectively. Different letter (small letters: $p < 0.05$; capital letters: $p < 0.01$) superscripts with boldface font in a column indicate significant differences among the different genotypes.
