# Supplementary material for: Genetic Polymorphisms of IGF1 and IGF1R Genes and Their Effects on Growth Traits in Hulun Buir Sheep
Source: Genes (Basel). 2022 Apr 9;13(4):666. doi: 10.3390/genes13040666 (PMC9031115; doi:10.3390/genes13040666)
Supplement: Supplementary file 1 [file genes-13-00666-s001.zip › Table S6.pdf]

**Table S6.** Associations for the haplotype combinations (block 1) of *IGF1R* gene with body weight traits and ADG traits in Hulun Buir sheep (mean ± SE, n = 229)

| Haplotype combination | Body weight (kg) | | | Average daily gain (ADG) (g) | | |
|---|---|---|---|---|---|---|
| | BW | WW | NBW | 0-4 ADG | 4-9 ADG | 0-9 ADG |
| H1H1 (71) TCTC | 4.15 ± 0.08 | 23.43 ± 0.85 | 32.32 ± 0.90 | 154.48 ± 6.43 | 61.37 ± 2.03 | 104.48 ± 3.20 |
| H1H2 (92) TCCT | 4.17 ± 0.07 | 22.79 ± 0.73 | 31.82 ± 0.78 | 148.61 ± 5.23 | 62.11 ± 1.92 | 102.33 ± 2.69 |
| H1H3 (22) TCCC | 4.36 ± 0.14 | 24.38 ± 1.5 | 33.25 ± 1.68 | 162.81 ± 11.68 | 61.58 ± 4.17 | 108.29 ± 6.17 |
| H2H2 (12) CTCT | 4.22 ± 0.12 | 24.89 ± 1.36 | 34.10 ± 1.32 | 163.23 ± 9.95 | 63.74 ± 3.41 | 110.30 ± 4.55 |
| H2H3 (32) CTCC | 4.24 ± 0.24 | 21.16 ± 1.33 | 29.93 ± 1.99 | 141.17 ± 10.26 | 60.31 ± 7.75 | 96.89 ± 7.22 |

BW = birth weight; WW = Weaning weight; NBW = weight at 9-mongth of age; 0-4 ADG, 4-9 ADG and 0-9 ADG represent the average daily weight gain before weaning, after weaning and from birth to 9-month of age, respectively. Different letter (small letters: $p < 0.05$; capital letters: $p < 0.01$) superscripts with boldface font in a column indicate significant differences among the different genotypes.
