# Supplementary material for: Genetic Polymorphisms of IGF1 and IGF1R Genes and Their Effects on Growth Traits in Hulun Buir Sheep
Source: Genes (Basel). 2022 Apr 9;13(4):666. doi: 10.3390/genes13040666 (PMC9031115; doi:10.3390/genes13040666)
Supplement: Supplementary file 1 [file genes-13-00666-s001.zip › Table S7.pdf]

Table S7. Associations for the haplotype combinations (block 1) of *IGF1R* gene with body size traits in Hulun Buir sheep (mean ± SE, n = 229)

Haplotype combination	Weaned body size (cm)			Body size of 9-month (cm)		
	WBH	WBL	WCG	NBH	NBL	NCG
H1H1 (71) TCTC	55.82 ± 0.61	57.27 ± 0.75	68.80 ± 0.91	64.13 ± 0.54	**67.22 ± 0.57**^{ab}	83.15 ± 0.92
H1H2 (92) TCCT	55.65 ± 0.57	56.58 ± 0.79	67.89 ± 0.79	63.63 ± 0.43	**66.00 ± 0.51**^{ab}	82.71 ± 0.79
H1H3 (22) TCCC	57.80 ± 1.01	58.95 ± 1.28	68.77 ± 1.53	63.07 ± 0.74	**68.91 ± 1.32**^{a}	83.98 ± 1.50
H2H2 (12) CTCT	56.77 ± 1.07	58.36 ± 1.30	69.61 ± 1.36	63.63 ± 0.85	**67.50 ± 0.99**^{ab}	85.83 ± 1.53
H2H3 (32) CTCC	55.33 ± 1.43	56.13 ± 1.42	66.63 ± 1.54	63.25 ± 1.47	**65.04 ± 1.39**^{b}	81.29 ± 2.48

WBH, WBL and WCG represent the body height, body length and chest girth measured at weaning respectively; NBH, NBL and NCG represent the body height, body length and chest girth measured at 9-mongth of age respectively. Different letter (small letters: $p < 0.05$; capital letters: $p < 0.01$) superscripts with boldface font in a column indicate significant differences among the different genotypes.
