# Supplementary material for: Genetic Polymorphisms of IGF1 and IGF1R Genes and Their Effects on Growth Traits in Hulun Buir Sheep
Source: Genes (Basel). 2022 Apr 9;13(4):666. doi: 10.3390/genes13040666 (PMC9031115; doi:10.3390/genes13040666)
Supplement: Supplementary file 1 [file genes-13-00666-s001.zip › Table S8.pdf]

Table S8. Associations for the haplotype combinations (block 2) of *IGF1R* gene with body weight traits and ADG traits in Hulun Buir sheep (mean ± SE, n = 229)

Haplotype combination	Body weight (kg)			Average daily gain (ADG) (g)		
	BW	WW	NBW	0-4 ADG	4-9 ADG	0-9 ADG
H4H4 (26) CGCG	**3.90 ± 0.14^{b}**	**20.98 ± 1.43^{BC}**	**30.02 ± 1.45^{bc}**	**137.77 ± 10.62^{ab}**	**62.36 ± 2.44^{A}**	**97.29 ± 5.10^{B}**
H4H5 (84) CGTG	**4.21 ± 0.07^{ab}**	**22.51 ± 0.80^{ABC}**	**31.61 ± 0.85^{ab}**	**146.56 ± 5.95^{ab}**	**62.65 ± 2.09^{A}**	**101.56 ± 3.00^{AB}**
H4H6 (11) CGCA	**4.07 ± 0.23^{ab}**	**19.16 ± 1.29^{C}**	**26.15 ± 1.39^{c}**	**123.39 ± 9.64^{b}**	**48.11 ± 5.35^{B}**	**82.44 ± 4.92^{C}**
H5H5 (74) TGTG	**4.24 ± 0.08^{ab}**	**25.14 ± 0.81^{A}**	**33.92 ± 0.84^{ab}**	**165.56 ± 5.81^{a}**	**60.72 ± 2.00^{A}**	**109.61 ± 2.86^{AB}**
H5H6 (34) TGCA	**4.33 ± 0.10^{a}**	**24.70 ± 1.02^{AB}**	**34.39 ± 1.18^{a}**	**165.80 ± 7.83^{a}**	**67.13 ± 3.85^{A}**	**112.58 ± 4.29^{A}**

BW = birth weight; WW = Weaning weight; NBW = weight at 9-mongth of age; 0-4 ADG, 4-9 ADG and 0-9 ADG represent the average daily weight gain before weaning, after weaning and from birth to 9-month of age, respectively. Different letter (small letters: $p < 0.05$; capital letters: $p < 0.01$) superscripts with boldface font in a column indicate significant differences among the different genotypes.
