# Supplementary material for: Genetic Polymorphisms of IGF1 and IGF1R Genes and Their Effects on Growth Traits in Hulun Buir Sheep
Source: Genes (Basel). 2022 Apr 9;13(4):666. doi: 10.3390/genes13040666 (PMC9031115; doi:10.3390/genes13040666)
Supplement: Supplementary file 1 [file genes-13-00666-s001.zip › Table S9.pdf]

Table S9. Associations for the haplotype combinations (block 2) of *IGF1R* gene with body size traits in Hulun Buir sheep (mean ± SE, n = 229)

Haplotype combination	Weaned body size (cm)			Body size of 9-month (cm)		
	WBH	WBL	WCG	NBH	NBL	NCG
H4H4 (26) CGCG	**53.90 ± 1.10^{B}**	**54.21 ± 2.13^{B}**	**66.35 ± 1.48^{ab}**	**62.08 ± 0.72^{BC}**	**65.04 ± 0.90^{C}**	**80.73 ± 1.40^{B}**
H4H5 (84) CGTG	**55.23 ± 0.63^{AB}**	**56.60 ± 0.71^{AB}**	**67.77 ± 0.88^{ab}**	**63.70 ± 0.50^{AB}**	**65.89 ± 0.56^{BC}**	**81.79 ± 0.85^{AB}**
H4H6 (11) CGCA	**54.55 ± 1.51^{AB}**	**52.95 ± 1.44^{B}**	**64.36 ± 1.55^{b}**	**60.45 ± 1.00^{C}**	**63.14 ± 0.93^{C}**	**76.23 ± 1.92^{C}**
H5H5 (74) TGTG	**57.24 ± 0.56^{A}**	**58.82 ± 0.71^{A}**	**69.93 ± 0.84^{a}**	**64.49 ± 0.51^{A}**	**67.96 ± 0.56^{AB}**	**85.86 ± 0.90^{A}**
H5H6 (34) TGCA	**57.57 ± 0.73^{A}**	**59.15 ± 0.79^{A}**	**69.69 ± 1.07^{a}**	**64.34 ± 0.62^{AB}**	**69.18 ± 0.90^{A}**	**85.90 ± 1.02^{A}**

WBH, WBL and WCG represent the body height, body length and chest girth measured at weaning respectively; NBH, NBL and NCG represent the body height, body length and chest girth measured at 9-mongth of age respectively. Different letter (small letters: $p < 0.05$; capital letters: $p < 0.01$) superscripts with boldface font in a column indicate significant differences among the different genotypes.
